# Continuous High-Dose (6 mg) vs. Low-Dose (3 mg) Intravitreal Ganciclovir for Cytomegalovirus Retinitis After Haploidentical Hematopoietic Stem Cell Transplantation: A Randomized Controlled Study

**DOI:** 10.3389/fmed.2021.750760

**Published:** 2021-12-24

**Authors:** Wei-Bin Chen, Ze Long, Jing Hou, Heng Miao, Ming-wei Zhao

**Affiliations:** ^1^Department of Ophthalmology and Clinical Center of Optometry, Peking University People's Hospital, Beijing, China; ^2^Eye Diseases and Optometry Institute, Peking University People's Hospital, Beijing, China; ^3^Beijing Key Laboratory of Diagnosis and Therapy of Retinal and Choroid Diseases, Beijing, China; ^4^College of Optometry, Peking University Health Science Center, Beijing, China

**Keywords:** high dose, intravitreal ganciclovir, treatment duration, cytomegalovirus retinitis, haploidentical hematopoietic stem cell transplantation

## Abstract

**Purpose:** To evaluate the safety and efficacy of continuous high-dose (6 mg) intravitreal ganciclovir injections (IVG) for cytomegalovirus (CMV) retinitis (CMVR) after haploidentical hematopoietic stem cell transplantation (Haplo-HSCT), and to explore factors that may influence the treatment procedure.

**Design:** Prospective, randomized, single-blinded, positive-controlled, interventional, comparative study.

**Methods:** A total of 22 patients with CMVR (32 eyes) were randomized to either high-dose group (IVG 6 mg weekly) or low-dose group (IVG 3 mg given twice weekly for 2 weeks as induction phase and weekly thereafter as maintenance phase). Patients who were recorded any positive CMV DNAemia or other active CMV diseases and needed systemic anti-CMV treatment during the study period were excluded. The vision outcome, variables of the treatment procedure, and incidence of complication and CMVR recurrence were analyzed and compared. Logistic regression was applied to determine the factors that may have an impact on the treatment process at baseline.

**Results:** Compared to the low-dose group, the high-dose group resulted in a median of two less intravitreal injections (4 vs. 6 times, respectively, *P* = 0.016), while the rate of vision stability or improvement (81.2 vs. 87.5%), the incidence of complication (6.2 vs. 18.8%), and CMVR recurrence (12.5% vs. 6.2%) were similar (all *P* > 0.05). No drug-related toxicity was observed. Initial aqueous CMV-DNA load (OR: 6.872, 95% CI: 1.335–35.377, *P* = 0.021) and extension of lesion (OR: 0.942, 95% CI: 0.897 to .991, *P* = 0.020), but not dosing regimen (*P* = 0.162), were predictors of the treatment duration.

**Conclusions:** Continuous high-dose regimen was well tolerated and resulted in less intravitreal injections, with similar vision outcomes and safety profiles. The clinical course of CMVR after Haplo-HSCT was determined by its own nature at baseline and could not be modified by treatment protocols under consistent immune background.

## Introduction

Haploidentical hematopoietic stem cell transplantation (Haplo-HSCT) expanded the selection range of donors but T-cell repletion and depletion approach around the procedure and subsequent immunomodulatory therapy altogether increased the risk of cytomegalovirus (CMV) disease after Haplo-HSCT (1). With the growing number of patients receiving Haplo-HSCT in China, cytomegalovirus retinitis (CMVR) is now becoming more and more common in routine ophthalmic clinics (2).

In previous studies, we had preliminarily established a treatment protocol for patients with CMVR after Haplo-HSCT (3–5). By continuously monitoring aqueous CMV-DNA load and interleukin-8 (IL-8) as biomarkers, the median number of the intravitreal injections of ganciclovir (IVG) was lowered by 2 times, and was proved to be safe and efficient (4). In these studies, 3 mg ganciclovir was used for each injection and the protocol requires an induction phase with an IVG twice a week, followed by a maintenance phase during which injections were given weekly (low-dose protocol). Patients after Haplo-HSCT usually have complex systemic comorbidities and situations (e.g., low platelet count, limited mobility, economic issues), that might make frequent injections not feasible, especially during the induction phase. Using an initial high dose of ganciclovir with 6 mg, Qian, etc. (6, 7) omitted the induction phase and the number of injections seemed to be fewer than low-dose treatment protocols. Besides, as viral resistance was encountered, a higher dose of 4 to 5 mg of ganciclovir for each injection was recommended during the maintenance phase (8). Considering the fact that increasing the total drug exposure (measured as average concentration area under the curve [AUC_0−24_]) improved the efficacy of ganciclovir for the maintenance treatment of CMVR, it is rational to extrapolate that continuous higher dose of IVG would be associated with increased efficacy and reduced the number of injections.

Accordingly, we conducted a prospective randomized study to compare the safety and efficacy of continuous IVG of 6 mg (high-dose protocol) vs. low-dose protocol to optimize the treatment options, maximize residual visual function, and reduce the treatment burden for patients with CMVR after Haplo-HSCT.

## Materials and Methods

### Study Design

This is a prospective, single-center, single-blinded, randomized-controlled, interventional, comparative study in patients with CMVR after Haplo-HSCT. The study was conducted from February 2016 to October 2020, at the Department of Ophthalmology, Peking University People's Hospital. The study protocol adhered to the tenets of the Declaration of Helsinki and was approved by the institutional review board of Peking University People's hospital under grant No. 2018PHB196-01.

### Setting and Patients

The off-label use of ganciclovir and its potential risks and benefits were discussed in detail with all patients, and written formal consents were acquired from each patient. Based on experience, we expected that a high-dose protocol would result in at least two less injections with a standard deviation of around 2 times (4–7). Using an α of 0.05 and power of 80%, a sample of 16 per group would be needed to detect two less injections between groups. The inclusion criteria included patients who had (1) history of Haplo-HSCT before CMVR diagnosis; (2) presence of suggestive fundus features of CMVR (any combination of the following retinal characteristic features found by an experienced ophthalmologist using indirect ophthalmoscopy: pale necrotic retina with focal areas of hemorrhage in a sectorial distribution spreading centrifugally along vascular arcades and vascular sheathing); (3) detection of CMV-DNA in the aqueous sample (>1 × 10^3^ copies/ml) using quantitative nucleic acid amplification testing (QNAT); (4) no other pathogens (herpes simplex virus, varicella-zoster virus, Epstein-Barr virus, toxoplasma gondi, fungi, etc.) that might cause similar ocular manifestation to CMV could be detected by a PCR from aqueous and (5) negative serum human immunodeficiency virus (HIV) antibody. The exclusion criteria included (1) patients who had other ocular diseases that could affect visual function (glaucoma, congenital keratopathy, macular disease, diabetic retinopathy, etc.); (2) history of intraocular surgeries; (3) patients who were recorded any positive serum CMV-DNA or coexistence of other active CMV disease besides CMVR during the study period, (4) HIV seropositive before transplantation, or (5) patients who were lost to follow up due to any other personal reasons.

### Treatment

Patients were randomized *via* a computer-generated randomization code with a 1:1 ratio into the high-dose group (Group 1) and the low-dose group (Group 2) in a masked fashion by H.M. For patients with bilateral CMVR, both eyes were included and adopted the same treatment protocol. The baseline values included age, gender, primary disease, human leukocyte antigen (HLA) locus match, duration of engraftment (defined as granulocyte survival), duration of lymphopenia (defined as peripheral lymphocyte <1.0 × 10^9^ /L) after Haplo-HSCT, duration from Haplo-HSCT to CMVR diagnosis, cumulative duration of CMV-DNAemia from Haplo-HSCT to CMVR diagnosis, and peak load of serum CMV-DNA after Haplo-HSCT.

Aqueous humor was extracted during the injection procedure before IVG. The aqueous CMV-DNA load was determined *via* QNAT, and the aqueous IL-8 level was measured using a cytometric bead array with flow cytometry (9).

For the high-dose group (Group 1), IVG 6 mg/0.1 ml was given weekly. For the low-dose group (Group 2), IVG 3 mg/0.1 ml was performed twice weekly for 2 weeks (4 times as the induction phase), followed by once a week as the maintenance phase.

When viral resistance occurred, as shown by aqueous CMV DNA load decreased <2 orders of magnitude, or IL-8 level decreased <1 order of magnitude right within the first 2 weeks after treatment initiation, intravitreal foscarnet (2.4 mg/0.2 ml) was added along with ganciclovir (3–5).

The treatment endpoint for both groups was defined as negative (<1.0 × 10^3^ copies/ml) CMV-DNA OR negative (<30 pg/ml) IL-8 in aqueous humor (4, 5).

When treatment ceased, the patients were followed up every 2 weeks during post-injection month 1, then monthly thereafter until post-injection month 6. Once recurrence occurred, treatment would be restarted using the same dosing regimen until lesion quiescence.

All patients underwent a comprehensive ophthalmological examination before each injection and at each post-injection follow-up. The best-corrected visual acuity (BCVA), intraocular pressure (IOP) (Goldman), inflammation in the anterior chamber/vitreous, and presence or absence of macular involvement were recorded at each examination. Ultrawide fundus photography (Optos Panoramic Ophthalmoscope P200MAAF 200Tx [Optos PLC, Dunfermline, Scotland]) and optical coherent tomography (OCT) (ZEISS CIRRUS HD-OCT4000 [Zeiss Meditec. Inc, Germany]) were also performed. The CMVR lesion area was determined using software Image-pro plus 6.0 (Media Cybernetics, CA, United States) and expressed as disc area (DA).

The serum CMV-DNA was determined at baseline and then monitored weekly. Additional measures included routine serum chemistry and hematology testing.

### Study Outcomes

The outcome measures included the number of intravitreal injections, treatment duration, the incidence of CMVR recurrence and complication, and vision stability or improvement rate at post-injection month 6.

Treatment duration was defined as the duration between the first and last injection. Recurrence was defined as an increase in the number of CMVR lesions, or the size of the preexisting lesions. Fundus and OCT images and were reviewed and measured independently by two ophthalmologists (H.M and M.W.Z) in a masked fashion. Any discrepancies in judgment were resolved through reassessment and discussion with a third ophthalmologist (J.H), also in a masked fashion.

### Statistical Analysis

The clinical characteristics of the patients, ocular findings, treatment regimen, and outcome data were analyzed using descriptive statistics. The Chi-square test with Fisher's exact correction was used to compare differences between categorical variables. Student's *t*-test and Mann–Whitney test were used for continuous variables between groups. Aqueous CMV-DNA load was plotted to time and Pearson correlation coefficient was calculated. The difference between the linear regression equations was evaluated by covariance analysis. Factors that might predict treatment duration were evaluated by univariate and multivariate logistic regression analyses. All analyses were performed using SPSS (version 24.0; IBM-SPSS, Chicago, IL, USA). All *P*-values were 2-sided and were considered statistically significant if < 0.05.

## Results

**Figure 1:**
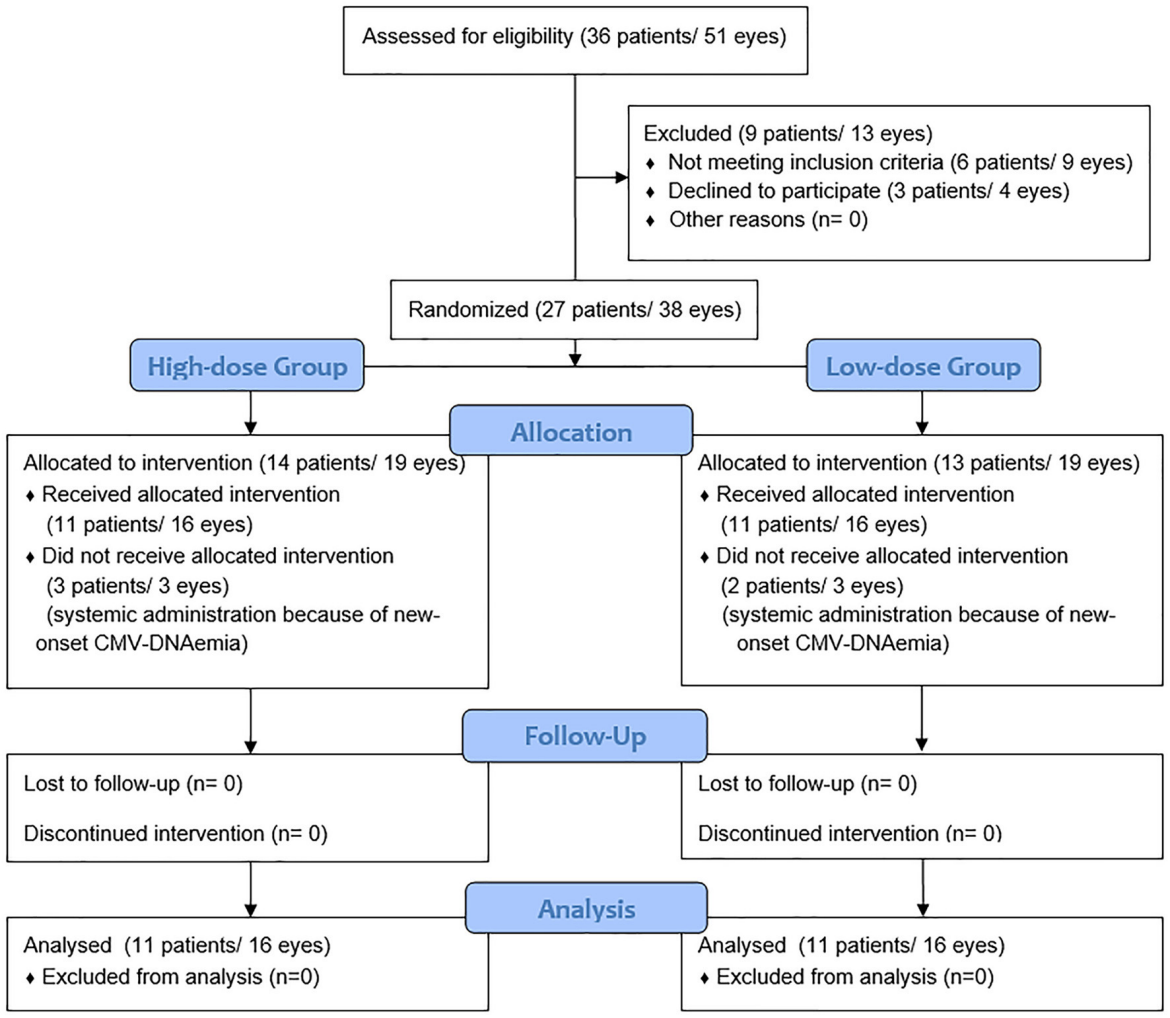
A flow diagram of the randomization procedure.

A total of 36 patients with CMVR was recruited in the study. Six patients were excluded because of CMV-DNAemia at baseline, and another three patients declined to participate. A total of 14 patients were randomized to the high-dose group (Group 1) and 13 patients were randomized to the low-dose group (Group 2). During the treatment, three patients in group 1 and two patients in group 2 were excluded because of new-onset CMV-DNAemia. Finally, the remaining 22 patients (32 eyes) meeting all criteria mentioned above completed this study and were analyzed and compared (Figure 1). The baseline characteristics are summarized in Table 1. The randomization achieved a good balance among the two treatment groups, and neither variable showed a significant difference between groups (all *P* > 0.05).

**Table 1:** General characteristics of the study population.

**Characteristic**	**Group 1**	**Group 2**	***P*** **-value**
Age, *y*, median (range)	28 (17–51)	29 (18–50)	0.948^a^
Gender			1.000^b^
Male	5 (45.5%)	4 (36.4%)	
Female	6 (54.5%)	7 (63.6%)	
Primary disease			0.254^b^
Acute myelocytic leukemia	2 (18.2%)	4 (36.4%)	
Acute lymphocytic leukemia	6 (54.5%)	4 (63.6%)	
Lymphoma	3 (27.3%)	1 (9.1%)	
Myelodysplastic syndromes	0 (0.0%)	2 (18.2%)	
Cumulative duration of CMV-DNA viremia from HSCT to CMVR diagnosis, *d*, median (range)	36 (11–85)	40 (18–234)	0.717^a^
Peak blood CMV-DNA before CMVR diagnosis, log_10_ (copies/ml)	4.26 ± 0.57	4.48 ± 0.35	0.286^c^
Interval between HSCT and CMVR diagnosis, *d*, median (range)	146 (34–367)	135 (91–354)	0.974^a^
Matching HLA locus	3 (3–6)	3 (3–4)	0.475^a^
Engraftment time, *d*, median (range)	14 (10–18)	13 (12–20)	0.715^a^
Duration of lymphopenia, *d*, median (range)	116 (34–158)	88 (46–170)	0.718^a^
Laterality of diseased eyes			1.000^b^
Unilateral	6 (54.5%)	6 (54.5%)	
Bilateral	5 (45.5%)	5 (45.5%)	
BCVA at baseline, LogMAR	0.36 ± 0.38	0.27 ± 0.29	0.468^c^
Intraocular pressure at baseline, mmHg	15.5 ± 1.9	18.7 ± 7.6	0.118^c^
Active inflammation in anterior segment			1.000^b^
No	12 (75.0%)	12 (75.0%)	
Yes	4 (25.0%)	4 (25.0%)	
Macular involvement			1.000^b^
No	13 (81.3%)	12 (75.0%)	
Yes	3 (18.7%)	4 (25.0%)	
Lesion area, DA	55.4 ± 35.5	57.6 ± 52.8	0.895^c^
Aqueous IL-8 level, log_10_ (pg/ml)	2.46 ± 0.71	2.59 ± 0.45	0.590^c^
Aqueous CMV DNA load, log_10_ (copies/ml)	4.27 ± 0.80	4.68 ± 0.85	0.173^c^

*Group 1: high-dose group; group 2: low-dose group; HSCT, hematopoietic stem cell transplantation; CMV, cytomegalovirus; CMVR, cytomegalovirus retinitis; HLA, human leukocyte antigen; BCVA, best-corrected vision acuity; LogMAR, logarithm of the minimum angle of resolution; DA, disc area; IL, interleukin*.

a *: Two-sample Wilcoxon ranksum (Mann–Whitney) test*.

b *: Fisher's Exact Test*.

c *: Student's T-test*.

**Figure 2:**
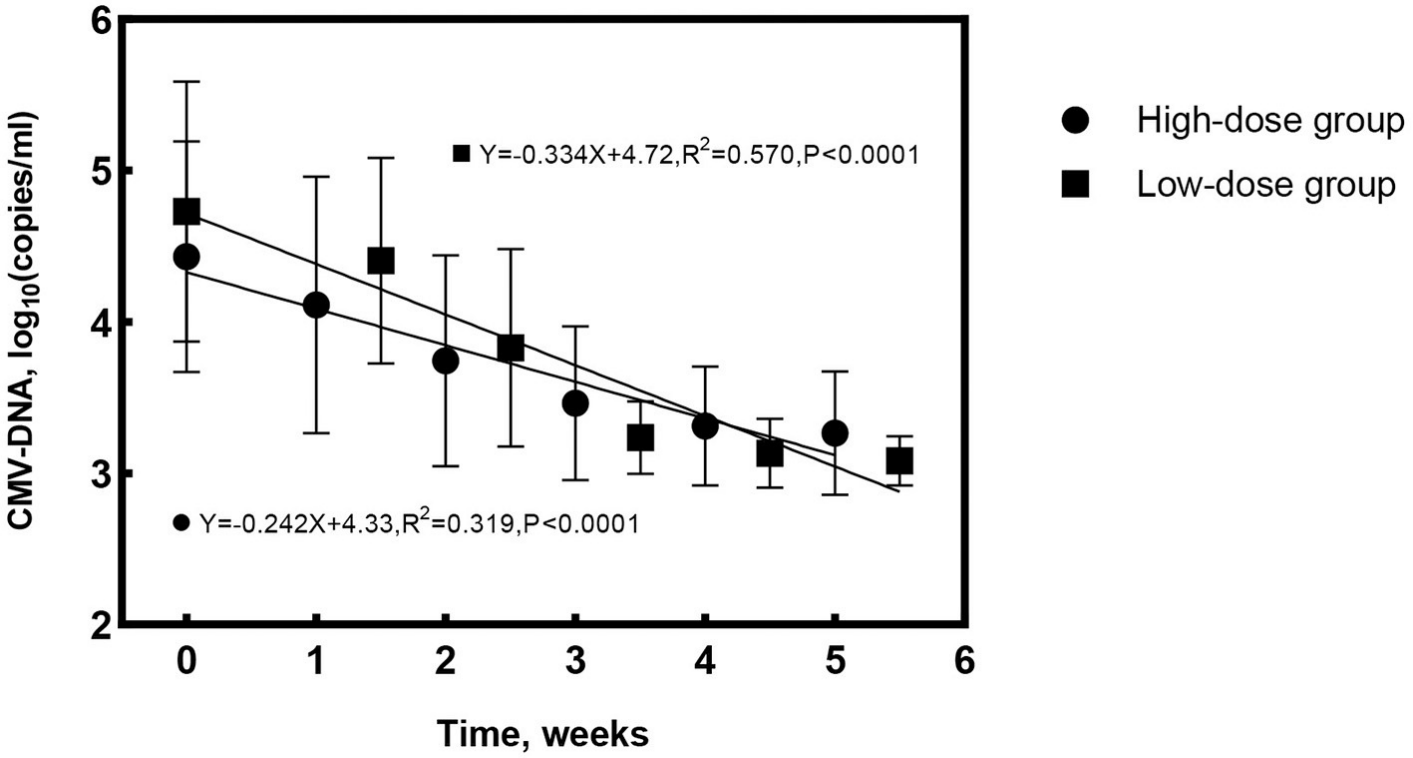
Changes of aqueous cytomegalovirus (CMV)-DNA load with time for the high-dose and low-dose groups. The rate of CMV-DNA clearance in aqueous was similar between groups (*F* = 2.067, *P* = 0.153).

**Figure 3:**
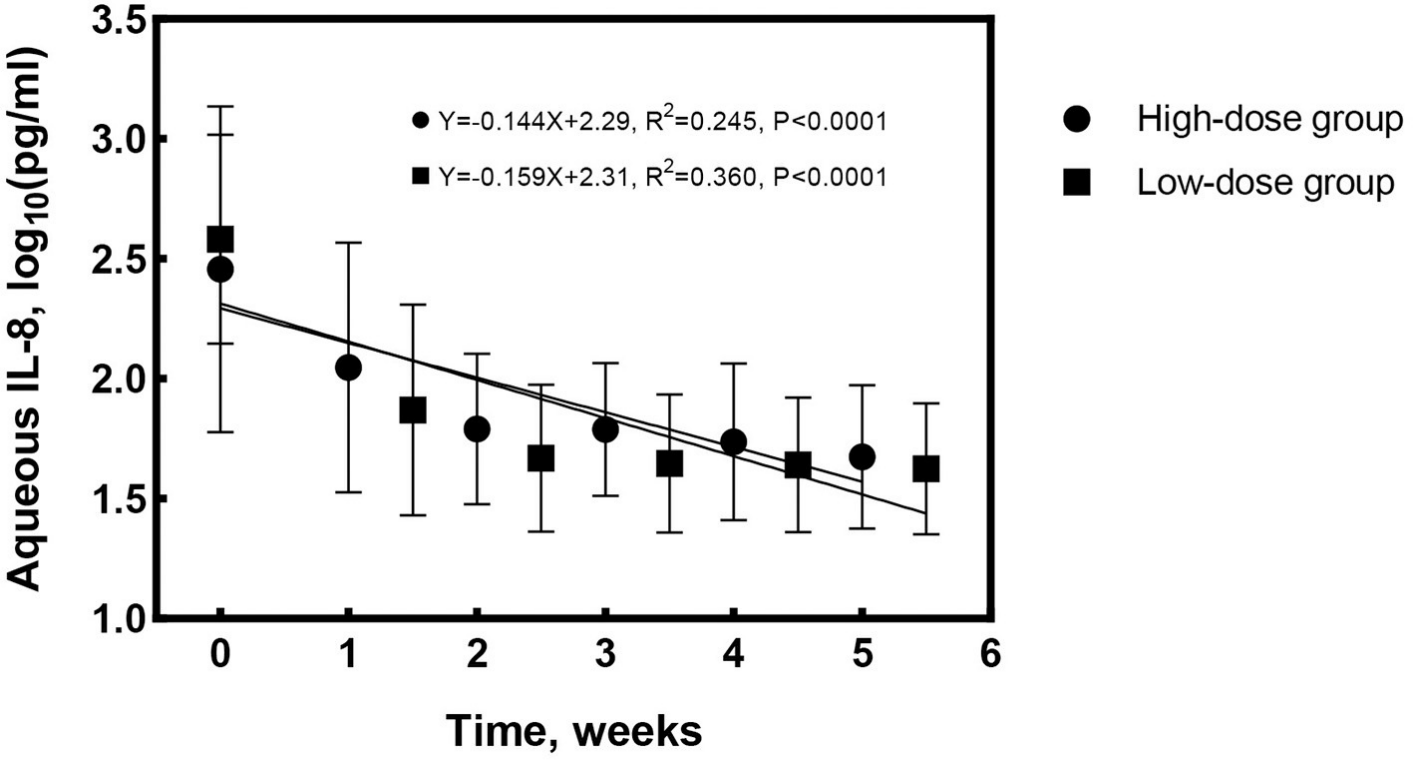
Changes of aqueous interleukin-8 (IL-8) level with time for the high-dose and low-dose groups. The decrease rate of aqueous IL-8 was similar between the groups (*F* = 0.081, *P* = 0.776).

At treatment cessation, the median number of IVG was significantly less in Group 1 compared to Group 2 (4 vs. 6 times, *P* = 0.016), but the treatment duration was almost the same (*P* = 0.282). The linear regression equation was applied to reflect the decreasing rate of aqueous CMV-DNA load and IL-8 by time in both groups. The correlation coefficients for aqueous CMV-DNA were 0.565 (*R*^2^ = 0.319, *P* < 0.0001, Figure 2) and 0.755 (*R*^2^ = 0.570, *P* < 0.0001, Figure 2) for Group 1 and Group 2, respectively. The covariance analysis did not find a significant difference between the groups (*F* = 2.067, *P* = 0.153). The decreasing rate of aqueous IL-8 level was also similar between the two groups (*F* = 0.081, *P* = 0.776, Figure 3). The correlation coefficients were 0.494 (*R*^2^ = 0.245, *P* < 0.0001, Figure 3) and 0.600 (*R*^2^ = 0.360, *P* < 0.0001, Figure 3) for Group 1 and Group 2, respectively. Take all the patients together, the median treatment duration was 24 days.

At the end of the six-month follow-up, a total of two eyes with vitreous hemorrhage and two eyes with rhegmatogenous retinal detachment were observed. Although not significant, the incidence of complications was higher in Group 2 compared to Group 1 (18.8 vs 6.2%, *P* = 0.600), which may result from the higher number of injections. Two eyes of rhegmatogenous retinal detachment were treated by pars plana vitrectomy and both eyes retained visual acuity after surgery. Two eyes of vitreous hemorrhage were followed up without specific management and the vision recovered after the hemorrhage was absorbed spontaneously. Two eyes in Group 1 (12.5%) and one eye in Group 2 (6.2%) suffered CMVR recurrence, which was also not statistically significant (*P* = 0.000, Table 2). After restarting the treatment, all eyes achieved quiescence at last. No drug or dose-related adverse event was observed. Vision improvement was achieved in 10/16 (62.5%) eyes in Group 1 and 9/16 (56.3%) eyes in Group 2. Three out of 16 eyes in group 1 and 5 out of 16 eyes in group 2 had a stable vision (*P* > 0.05, Table 2). The mean BCVA and IOP were also comparable between groups (both *P* > 0.05).

**Table 2:** Safety and efficacy profiles comparison between high-dose and low-dose group.

**Outcome**	**Group1**	**Group2**	***P*** **-value**
Times of intravitreal injection, median (range)	4 (2–8)	6 (4–8)	0.016^a^
Treatment duration, *d*, median (range)	21 (7–52)	26 (11–43)	0.282^a^
BCVA at month 6, LogMAR	0.30 ± 0.34	0.19 ± 0.27	0.314^b^
Intraocular pressure at month 6, mmHg	13.63 ± 2.78	14.00 ± 2.50	0.691^b^
Vision stability or improvement			1.000^c^
No	3 (18.8%)	2 (12.5%)	
Yes	13 (81.2%)	14 (87.5%)	
Interval between treatment initiation and vision stability, *d*, median (range)	23 (14–59)	26 (17–50)	0.933^a^
CMVR Recurrence			1.000^c^
No	14 (87.5%)	15 (93.8%)	
Yes	2 (12.5%)	1 (6.2%)	
Interval between local treatment cessation and recurrence, d	30, 34	98	0.221^a^
Complication			0.600^c^
No	15 (93.8%)	13 (81.2%)	
Yes	1 (6.2%)	3 (18.8%)	
State at the end of local treatment			0.920^c^
CMV DNA (–) and IL-8 (-)	5(31.3%)	6(37.5%)	
CMV DNA (–) and IL-8 (+)	7(43.7%)	6 (37.5%)	
CMV DNA (+) and IL-8 (–)	4(25.0%)	4(25.0%)	

*Group 1: high-dose group; Group 2: low-dose group; BCVA, best corrected vision acuity; LogMAR, logarithm of the minimum angle of resolution; CMVR, cytomegalovirus retinitis; IL, interkeukin*.

a *: Two-sample Wilcoxon rank-sum (Mann-Whitney) test*.

b *: Student's T-test*.

c *: Fisher's exact test*.

The impact of the baseline characteristics and dosing regimen on the treatment duration was evaluated by logistic regression analyses (Table 3). In the univariate regression, the baseline aqueous CMV-DNA load (OR: 3.031, 95% CI: 1.096–8.377, *P* = 0.033) and the lesion area (OR:0.961, 95% CI:0.931–0.992, *P* = 0.014) were the predictive factors for longer treatment duration (≥24 days), and the interval between Haplo-HSCT and CMVR diagnosis showed borderline significance (*P* = 0.074). When all factors were included in the multivariate regression model, only a smaller CMVR lesion area (OR:0.942, 95% CI:0.897–0.991, *P* = 0.020) and higher aqueous CMV-DNA load (OR:6.872, 95% CI: 1.335–35.377, *P* = 0.021) at baseline were associated with longer treatment duration (≥24 days). The treatment protocol did not influence the treatment duration (*P* = 0.162 in univariate analysis, and *P* = 0.506 in multivariate analysis).

**Table 3:** Univariate and multivariate logistic regression analyses of risk factors for treatment duration ≥24 days.

**Variable**	**Univariate analysis**		**Multivariate analysis**	
	**OR(95% CI)**	***P*** **-value**	**OR(95% CI)**	***P*** **-value**
Age	0.937 (0.864–1.016)	0.115	0.919 (0.813–1.039)	0.179
Gender	1.667 (0.407–6.818)	0.477	0.478 (0.060–3.810)	0.486
Primary disease	1.171 (0.536–2.557)	0.693	0.990 (0.311–3.149)	0.986
Matching HLA locus	0.878 (0.431–1.788)	0.720	0.665 (0.230–1.919)	0.450
Engraftment time	1.181 (0.940–1.485)	0.154	1.039 (0.746–1.449)	0.820
Duration of lymphopenia	1.005 (0.988–1.025)	0.506	1.005 (0.978–1.033)	0.704
Interval between HSCT and CMVR diagnosis	0.990 (0.979–1.001)	0.074	0.994 (0.980–1.007)	0.362
Cumulative duration of CMV viremia before CMVR diagnosis	0.989 (0.970–1.008)	0.263	0.987 (0.955–1.020)	0.442
Peak blood CMV-DNA before CMVR diagnosis	2.318 (0.501–10.726)	0.282	2.109 (0.254–17.526)	0.490
BCVA at baseline	0.329 (0.032–3.347)	0.348	1.181 (0.027–51.996)	0.931
Intraocular pressure at baseline	0.987 (0.871–1.118)	0.836	0.969 (0.828–1.134)	0.695
Inflammation in the anterior segment	1.000 (0.202–4.955)	1.000	0.351 (0.023–5.452)	0.455
macular involvement	1.444 (0.266–7.829)	0.670	0.949 (0.104–8.629)	0.963
Lesion area at diagnosis	0.961 (0.931–0.992)	0.014	0.942 (0.897–0.991)	0.020^a^
Aqueous IL-8 at diagnosis	0.867 (0.304–2.474)	0.789	1.473 (0.212–10.207)	0.695
Aqueous CMV DNA at diagnosis	3.031 (1.096–8.377)	0.033	6.872 (1.335–35.377)	0.021^a^
Treatment protocol	0.360 (0.086–1.506)	0.162	0.509 (0.070–3.721)	0.506

*OR, Odds ratio; CI, confidence interval; HLA, human leukocyte antigen; HSCT, hematopoietic stem cell transplantation; CMV, cytomegalovirus; CMVR, cytomegalovirus retinitis; BCVA, Best-corrected visual acuity; IL, interleukin*.

a *: statistically significant with value of p < 0.05*.

## Discussion

As the half-life of ganciclovir in non-vitrectomized eyes was about 18.8 h (10) and the 50% inhibitory concentration (IC_50_) of ganciclovir for CMV was around 1 mg/L (11), IVG of 2 mg given weekly as maintenance therapy was widely used for several kinds of herpetic necrotizing retinopathies. During the induction phase, a doubled-frequency was required to maximize the intraocular drug concentration quickly. Dozens of studies had proved the safety and efficacy of this regimen (classic protocol) (12–14), but several clinical problems remained. (1) Patients with CMVR after Haplo-HSCT usually have complex systemic comorbidities and situations, such as low platelet count, limited mobility, economic concerns, etc., that may make frequent injections not feasible or even dangerous, especially during the induction phase. (2) As all patients were CMV seropositive before Haplo-HSCT, all the patients with CMVR had experienced CMV-DNAemia before CMVR diagnosis and had received intravenous ganciclovir for treatment. Some of them even took prolonged low-dose oral ganciclovir for prophylaxis before inclusion, which might, in turn, increase the risk of CMV resistance when CMVR develops. (3) It is more difficult to monitor intraocular CMV-DNA load than blood because aqueous could only be collected during the injection procedure. Although CMV resistance could be determined as we described in earlier reports (3, 4), once developed, prolonged treatment would be expected and more problems would arouse. Concerning the fact that a single injection of 2 mg ganciclovir only results in borderline intraocular drug concentration since the 5th day after injection (10, 11), low level of ganciclovir may induce CMV resistance (15–17), and patients at immune-compromised state were prone to ganciclovir resistance (16, 18), the classic protocol needs to be improved to adapt to CMVR patients after Haplo-HSCT.

Qian et al. (7) used an initial high dose of ganciclovir to treat CMVR in HIV-negative patients, in which the induction phase was replaced by high dose ganciclovir injection given weekly, and the mean number of injections was lowered to about 5 times compared to former studies. Besides, Lalezari et al. (19) found that, when given orally, the total exposure (measure as average serum AUC_0−24_ of ganciclovir) rather than peak level correlated best with the time to CMVR progression in HIV positive patients. Short periods of high extracellular concentration of drug as provided by IVG 2–3mg do not appear to be necessary for efficacious maintenance treatment. Thus, it is rational to believe that continuous high-dose IVG would show better performance through reducing the number of injections by removing the induction phase, avoiding CMV resistance during the maintenance phase, and finally resulting in shorter treatment duration and lower expenses.

A higher dose of ganciclovir for intravitreal injection had been described by several authors, ranging from 4 to 6 mg, and no dose-related ocular adverse events were reported (6–8, 20). The crystallization of ganciclovir in the vitreous cavity or syringe had been described in several cases, but its concentration ranged from 2.5 mg/0.05 ml to 4 mg/0.04 ml, which represented the influence of pH or temperature on the physical nature of ganciclovir rather than concentration itself (21, 22). Repeated intraocular crystallization did not seem to cause any harm to ocular tissues either (21). As the half-life period of ganciclovir is around 18.8 h, drug accumulation was considered highly unlikely by weekly injection (10). In our study, no drug or dose-associated adverse event was observed during the whole study period in both groups, which proved the safety of a continuous high-dose regimen.

In our previous studies, by incorporating aqueous humor IL-8 level into the criteria of CMVR treatment decision, two less injections (from 8 to 6 times) could be achieved safely and effectively (4). And in this study, using the same decision criteria, weekly high-dose (6 mg) IVG without induction phase further reduced the median number of injections from 6 to 4 times. At the end of the 6-month follow-up, the vision outcome, incidence of complications, and recurrence of CMVR were all comparable between the groups, which indicated that a continuous high-dose regimen could achieve in less injections safely, while the efficacy was the same as the low-dose regimen.

Another important finding was that, despite different dosing and frequency of IVG, the treatment duration did not show any difference between groups. When aqueous CMV-DNA and IL-8 were plotted to time, the slopes showed no significant difference, either. Multivariate logistic regression further demonstrated that initial aqueous CMV-DNA load and lesion area rather than dosing protocol were factors that influence the duration of treatment. Thus, it seemed that a continuous high-dose regimen was capable of reducing the total number of injections, but cannot shorten the clinical course of CMVR when treatment started. The natural course of CMVR after Haplo-HSCT was determined by its own nature and could not be modified by dosing or frequency of treatment, which was much more like a self-limited disease under certain circumstances (see discussion below).

Although the association between higher level of initial aqueous CMV-DNA load and longer treatment duration had been reported by several authors (7, 14), it was interesting to find that a larger lesion area at baseline indicated a shorter time to achieve disease quiescence in this study. In the literature, the relationship between lesion area and treatment duration (expressed as the number of injections) was controversial. Aniruddha et al. (12) reported a positive correlation between the size of the lesion and the number of injections required. Jeon et al. (14) also found a positive association between the size of the involved retina and the number of IVG in a simple regression analysis, but multiple regression analysis denied that association. The discrepancy mainly resulted from the design and the inclusion criteria of these studies, because most of them were retrospective and the patients were included irrespective of the presence of CMV-DNAemia during follow up, which indicated poorer general condition or significant immune background deviation (18, 23, 24), and additional intravenous or oral ganciclovir was needed. In this study, patients with the presence of CMV-DNAemia or other active CMV diseases were all excluded to minimize the deviation of their immune background throughout the study period. Thus, factors apart from ophthalmologic concerns that may interfere with the treatment process were excluded as far as possible. As supported by larger studies of the course of CMVR in HIV-positive patients, more extensive areas of retinitis resulted from delayed diagnosis (25). Considering the phenomenon that clinical course of CMVR after Haplo-HSCT was determined by its own nature at baseline and did not vary between different dosing protocols, for patients without significant immune background deviation, a larger lesion area suggested that the course of the disease was coming to an end, and less time was needed to achieve disease quiescence.

The strength of our results benefits from the prospective, randomized, controlled design and relatively stable immune background during the treatment and follow-up process. But several limitations need to acknowledge. First, the sample size is small due to the low incidence of such disease, which was reported to be 2.3% 1 year after Haplo-HSCT (26), and both eyes of patients with bilateral CMVR had to be included at the same time. This might cover some minor significant associations between factors. Considering the fact that every variable at baseline, including laterality, was distributed evenly, our results were still reliable and clear. Second, patients with CMV-DNAemia occurring at any time during the whole procedure were excluded from this study to keep the immune background consistent. Thus, the results from this study must be interpreted with caution, as the deviation of immune status, represented by the occurrence of CMV-DNAemia, may potentially affect the treatment procedure and outcome. Further studies are needed to address this issue.

In summary, continuous weekly intravitreal injection of 6 mg ganciclovir was well tolerated and resulted in less intravitreal injections compared with 3 mg-induction-and-ntemaintenance protocol, with similar vision outcome and safety profiles. The clinical course of CMVR after Haplo-HSCT was determined by its own nature at baseline, such as initial aqueous CMV-DNA load and lesion extension, and could not be modified by treatment protocols under consistent immune background.

## Data Availability Statement

The raw data supporting the conclusions of this article will be made available by the authors, without undue reservation.

## Ethics Statement

The studies involving human participants were reviewed and approved by Institutional Review Board of the Peking University People's Hospital. Written informed consent to participate in this study was provided by the participants' legal guardian/next of kin.

## Author Contributions

W-BC, HM, and M-wZ designed this study and wrote this article. JH, HM, and M-wZ collected and measured data. W-BC and ZL analyzed data. All authors discussed the results and commented on the manuscript.

## Funding

This work was supported by the National Key R&D Program of China under Grant No. 2020YFC2008200, the National Natural Science Foundation of China under Grant No. 81800847, and the tenth Academic Star of Peking University People's Hospital under Grant No. RS2018-05.

## Conflict of Interest

The authors declare that the research was conducted in the absence of any commercial or financial relationships that could be construed as a potential conflict of interest.

## Publisher's Note

All claims expressed in this article are solely those of the authors and do not necessarily represent those of their affiliated organizations, or those of the publisher, the editors and the reviewers. Any product that may be evaluated in this article, or claim that may be made by its manufacturer, is not guaranteed or endorsed by the publisher.
